# The Role of Manual Therapy in Patients with COPD

**DOI:** 10.3390/healthcare7010021

**Published:** 2019-02-01

**Authors:** Stephanie Clarke, Prue E. Munro, Annemarie L. Lee

**Affiliations:** 1 Department of Physiotherapy, Eastern Health, 5 Arnold Street, Box Hill, Victoria 3128, Australia; Stephanie.Clarke@easternhealth.org.au; 2 Department of Physiotherapy, School of Primary and Allied Health Care, Monash University, Victoria 3199, Australia; Prue.Munro@monash.edu; 3 Institute for Breathing and Sleep, Austin Health, 145 Studley Road, Heidelberg, Victoria 3084, Australia; 4 Physiotherapy, Rehabilitation, Nutrition and Sport, College of Science, Health and Engineering, La Trobe University, Bundoora, Victoria 3088, Australia

**Keywords:** chronic obstructive pulmonary disease, manual therapy, exercise tolerance, pain, musculoskeletal dysfunction

## Abstract

Chronic obstructive pulmonary disease (COPD) is a respiratory condition associated with altered chest wall mechanics and musculoskeletal changes. In this narrative review, we describe the underlying musculoskeletal abnormalities in COPD, the reasons for applying manual therapy techniques, their method of application and clinical effects. A variety of manual therapy techniques have been applied in individuals with COPD, including soft tissue therapy, spinal and joint manipulation and mobilisation, and diaphragmatic release techniques. These have been prescribed in isolation and in conjunction with other treatments, including exercise therapy. When applied in isolation, transient benefits in respiratory rate, heart rate and symptoms have been reported. Combined with exercise therapy, including within pulmonary rehabilitation, benefits and their corresponding clinical relevance have been mixed, the extent to which may be dependent on the type of technique applied. The current practical considerations of applying these techniques, including intense therapist–patient contact and the unclear effects in the long term, may limit the broad use of manual therapy in the COPD population. Further high quality research, with adequate sample sizes, that identifies the characteristic features of those with COPD who will most benefit, the optimal choice of treatment approach and the longevity of effects of manual therapy is required.

## 1. Introduction

Chronic obstructive pulmonary disease (COPD) is characterised by dyspnoea and fatigue, which are linked to reduced exercise tolerance and impaired health-related quality of life (HRQOL) [1,2]. Extrapulmonary manifestations of this condition, including musculoskeletal dysfunction, have been described in COPD [3], originating from respiratory pathophysiology and changes in musculoskeletal structures. Clinically, individuals with COPD may present with postural abnormalities, musculoskeletal pain or other symptoms, which warrant further treatment and lend support to the possible role of manual therapy. This narrative review explores the origins of the musculoskeletal changes that are targeted by these techniques, the types of techniques, their methods of application and effects and the clinical applicability to the COPD population. 

## 2. Musculoskeletal Changes Associated with COPD 

To understand the role of manual therapy in this population, it is important to evaluate the musculoskeletal changes which occur in COPD. This condition is characterised by respiratory muscle dysfunction. The respiratory muscle alterations are largely influenced by the development of pulmonary hyperinflation, which forces the diaphragm to operate at non-optimal lengths [4]. This results in a reduced contractile force relative to lung volume [5,6]. Specifically, hyperinflation is believed to impose a passive increase in chest wall rigidity and to alter chest wall mechanics with reduced chest wall compliance. In addition, the increase in airway resistance and airflow limitation encourages greater recruitment of the accessory muscles of respiration. Although the accessory respiratory muscles adapt to this increased load [7], the dual purpose of these muscles in upper limb and neck motion imposes limitations in upper limb based functional tasks [8]. To some extent, this reduced use contributes to increased respiratory muscle tightening, joint stiffness and increased work of breathing [8].

These changes in chest wall rigidity and muscular function may be linked to the postural alterations and changes in rib cage configuration noted in COPD [9,10]. Vertebral deformities have been observed [11,12], with an increase in thoracic kyphosis compared to aged-matched healthy individuals [10,11,12,13,14]. The majority of changes are located in the mid-thoracic and the thoraco-lumbar junction [11,15], which may be related to the prevalence of osteoporosis (9 to 69%) in COPD [16] and its association with kyphotic deformities [17]. Other observed postural changes in COPD include an increase in head and cervical protraction, reduced shoulder range of motion [18,19] and a corresponding increase in scapula elevation and upward rotation [20], exacerbated by the body positions adopted to relieve dyspnoea in COPD [21]. 

Other contributing factors include ageing and osteoarthritis. As a result of ageing, chest wall compliance is reduced, due to decreased thickness of the intervertebral discs and increased stiffness in the ribs [22]. Osteoarthritis affects between 12% and 74% of people with COPD [23]. In the chest wall, osteoarthritic changes have been documented in the manubriosternal joint, costovertebral and costotransverse joints [24,25]; this may account for reduced thoracic motion and hypomobility in the spinal, costal and sternal joints noted in COPD [18,26,27,28]. 

Adverse clinical consequences of these musculoskeletal changes or altered chest wall mechanics in COPD have been explored. Changes in chest wall mechanics have been linked to exercise-limiting dyspnoea in COPD [5]. Concurrent with these changes is peripheral muscle dysfunction, with muscle disuse atrophy, changes in muscle fibre type, reduced oxidative capacity and corresponding reduction in strength and endurance [29]. This combination has a negative influence on mobility and function, with lower levels of exercise capacity and physical activity [29]. An early study illustrated that reduction in thoracic spine mobility was linked to lower lung function, with decreases in forced vital capacity (FVC) and forced expiratory volume in one second (FEV_1_) [30]. More recent examination found that those with thoracic hyperkyphosis and COPD have less diaphragmatic mobility compared to those without with postural change, although the implications for this on respiratory function and symptoms are unclear [31]. In some individuals, postural changes have been associated with musculoskeletal pain [9,10]. In particular, thoracic vertebral deformities, with narrowed disc spaces and costotransverse joint arthropathy have been identified as predictors of trunk pain in COPD [10]. In addition, osteoarthritis has been hypothesised as a possible cause of or contributing factor to musculoskeletal pain, although the exact mechanism has not been explored [32]. 

Given this range of confirmed or possible clinical impact of these musculoskeletal changes in COPD, there is support for the use of treatment interventions that target thoracic joints and the soft tissues, and which have the potential to address joint stiffness and postural and muscular changes and to alleviate pain. 

## 3. Definition of Manual Therapy 

Manual therapy is a type of treatment intervention using specific hands-on techniques, including but not limited to manipulation and mobilisation of the soft tissue and joint structures, and is delivered by skilled practitioners [33]. They are applied for the purpose of improving tissue extensibility, range of motion, inducing relaxation, mobilising, manipulating, modulating pain, improving contractile and non-contractile tissue repair, extensibility or stability, reducing soft tissue swelling, inflammation or restrictions, facilitating movement and improving function. 

Manual therapy has been used to address the musculoskeletal anomalies in COPD. Clinical studies using these techniques are shown in Table 1. Techniques have been applied to the cervical and thoracic vertebral and costal joints, as well as the anterior and posterior chest wall/trunk and upper limb muscles. Manipulation and mobilisation involves the graded delivery of low or high velocity low amplitude joint mobilisation to the thoracic intervertebral, costovertebral and costotransverse joints at identified regions of spinal stiffness or observation of abnormalities in the paravertebral tissues [34]. When applied in COPD, the underlying reasoning is that these mobilisation strategies will temporarily improve chest wall compliance, addressing one element contributing to chest wall rigidity. Soft tissue therapy techniques that have been applied in COPD include effleurage, trigger points, cross fibre frictions and myofascial release [35,36,37]. The techniques are believed to encourage vasodilation and smooth muscle relaxation and to increase blood flow, which may assist in improving range of motion, reducing pain and promoting changes at the tissue level [38]. 

## 4. Application of Manual Therapy in COPD 

Despite limited publication in this area, current evidence highlights the potential benefit of manual therapy in COPD. Within these studies, from a practical perspective, techniques have been generally applied in combination, and the effects have been observed when administered in a single treatment session, over a series of treatments and in conjunction with other management, such as pulmonary rehabilitation. In the majority of cases, the therapist directly applies the techniques, with the occasional use of instrument-assisted spinal manipulation [39]. 

One of the earliest studies [40] investigated the immediate effects of a single 20 min treatment session, using seven standardised osteopathic manipulative techniques (e.g., soft tissue therapy to the paraspinal muscles, rib raising, sub-occipital decompression, thoracic inlet myofascial release, and pectoral traction). Thirty minutes following the treatment, respiratory function (inspiratory capacity, residual volume (RV) and RV as a proportion of total lung capacity (TLC)) increased, but forced expiratory flow at 50% and 75% of vital capacity was reduced. This mixed impact on respiratory function makes it difficult to determine the direct benefit or harm of each of the individual techniques. A slightly longer single treatment session (45 min) to people with severe to very severe COPD consisted of manual therapy to the cervical and thoracic spinal joints, scapulo-thoracic mobilisation, rib raising and myofascial release [41]. The full treatment protocol is outlined in Table 1. In contrast to the earlier study [40], improvement in spirometry and respiratory muscle strength were noted immediately post treatment, as a well as a decrease in heart rate and respiratory rate, and lower perceptions of dyspnoea and fatigue [41]. Despite some evidence of benefit, these approaches have not gained clinical acceptance due to the small sample sizes and the limited duration of effect immediately following treatment.

There have been studies which have compared the effects of either soft tissue therapy alone; in combination with spinal manipulation; or soft tissue therapy, spinal manipulation and exercise therapy [34,35]. In each of these studies, a longer duration of treatment (ranging from four to eight weeks) was applied. The specific techniques used in each protocol are outlined in Table 1 and were generally applied over a series of treatment sessions. All three therapies (soft tissue, spinal manipulation and exercise) combined improved FVC beyond the defined minimal important difference that is considered clinically relevant for this measure, and this was perceived to be the result of a synergistic interaction between the interventions [36]. This may account for the reduced dyspnoea and improved exercise tolerance based on a modified form of the 6 min walking test with both soft tissue therapy and spinal manipulation with and without exercise [34]. A slight variation on this combination compared pulmonary rehabilitation, soft tissue therapy and pulmonary rehabilitation, and spinal manipulative therapy and pulmonary rehabilitation [35] with the manual therapy applied during weeks 4 to 12 of the pulmonary rehabilitation program. It has been well established that pulmonary rehabilitation improves symptoms, exercise capacity and quality of life in people with COPD, with no change in respiratory function [44]. However, this study demonstrated that the addition of soft tissue therapy and spinal manipulation to a pulmonary rehabilitation program significantly improved respiratory function (FVC) at 24 weeks, although there was no effect on functional exercise capacity, anxiety, depression or quality of life. The clinical value of improving FVC with these adjunctive treatments from a patient’s perspective is largely unclear [45]. Despite this, the reduction in dyspnoea, according to a valid and reliable measurement tool evident in one study, and increase in walking distance lends support to the possible advantages in selected outcomes. Although the evidence of some improvements suggests that these techniques may be helpful across the spectrum of COPD when applied using a uniform, structured approach, the benefits appear to be short term, with no long term follow up in these studies. 

An alternative approach was applied by Zanotti et al. [42] and was described as treatment to address any dysfunction observed in the thoracic outlet, spine, ribcage, thoracic and pelvic diaphragm, tentorium cerebelli, and cranio-sacral regions. Like Engel in two previous studies [34,35], this was applied during a pulmonary rehabilitation program. Greater improvements in exercise capacity and reduction in RV were noted compared to those attending pulmonary rehabilitation alone [42]. Compared to the uniform approach to treatment applied in previous studies [34,35], it is possible that by basing a treatment on assessment findings, this may enable a more directed treatment that is appropriate for each individual, and may account for improvement in this patient related outcome of exercise capacity.

In contrast to manual therapies focused on the thoracic spine and rib cage, an alternative focus is muscle release techniques. Diaphragm release techniques in people with clinically stable COPD have been investigated over a series of six sessions [43]. In this technique, manual contact is applied bilaterally to the underside of the 7th to 10th rib costal cartilages by a therapist, who applies a cephalic and lateral pull during inspiration, accompanying rib elevation. This pressure is maintained during expiration, with the therapist deepening contact towards the inner costal margin. Depth of contact is increased in subsequent breaths. It is hypothesised that this contact allows cranial-directed traction of the ribs, along with lengthening of the diaphragm near its insertion around the anterior costal margin, produced by the compression of the diaphragm fibres in this area. The lengthening of the diaphragm, in combination with the therapist-facilitated movement of the ribs, is thought to improve the mobility of the diaphragm. The results found a cumulative increase in diaphragm mobility (measured by ultrasound) by 18 mm, and an improvement in inspiratory capacity. In addition, functional exercise capacity measured by the 6 min walk distance (6MWD) improved by 15 m with diaphragmatic release, compared to a deterioration of 6 m in the control group [43]. As inspiratory capacity is closely linked to the ventilatory demands of exercise [46], and the improvement in 6MWD of 22 m is within close proximity of the clinically relevant minimal important difference of this outcome in people with COPD [47], this suggests a potential clinical benefit. The observation of only cumulative effects highlights the need for repeated treatment. 

## 5. Clinical Considerations and Limitations of Manual Therapy in COPD 

In view of these findings, it is important to consider the practical aspects of these approaches and their clinical relevance. Beyond muscle soreness that resolved within 24–48 h, no adverse events have been noted with therapist-directed manual therapy [34,39]. Patient tolerability of repeated manual therapy intervention is important as it is likely required for clinical improvement. 

It is essential to consider the time and therapist-dependent effort involved to deliver the treatments, particularly when the benefits may only be achieved through multiple applications over the course of a number of weeks. Each of these treatments is labour and time intensive. With the broad prevalence of COPD, this is likely to limit their use in clinical practice. The additional element of treatment burden on patients, particularly those already engaged with pulmonary rehabilitation or other therapeutic approaches with known benefit, and financial cost to the patient should also be considered. 

The temporary improvements in respiratory function and symptoms following a single treatment session highlights that these techniques are targeting regions of musculoskeletal dysfunction. The diaphragmatic release technique is associated with improvement in diaphragmatic mobility, vital capacity and functional exercise tolerance when applied over a series of sessions [43]. Although only examined in one study, these findings support future examination of this technique, either applied independently or in conjunction with other types of manual therapy. The mixed results for symptoms of dyspnoea, functional exercise capacity, psychological symptoms, quality of life and lung function when manual therapy was applied as an adjunct to exercise therapy or pulmonary rehabilitation highlight important clinical practice points. With benefits in selected measures of lung function achieved using a mix of techniques, it is difficult to identify from the current studies whether there is a superior technique. The lack of consistency in improvement in functional exercise capacity with adopting a uniform approach to treatment [34,35], compared to selecting techniques based on an individual assessment [42], favours a patient-focused intervention. However, with the small number of participants included in each of these studies, further high quality clinical trials with sufficient sample sizes across multi-centre sites, and consistent use of valid and reliable outcome measures with blinded assessors is required to clarify this information. The precise mechanisms of action of each technique or combination of techniques is largely inferred. Establishing the effect on alternative measures of respiratory function, including chest wall expansion [48], may offer further insight into the mechanisms of action of each technique or combination of techniques. Finally, as the longest follow up period was 24 weeks in the current research [35], establishing the longer term effects of manual therapy in this population is necessary. 

It may be considered a strength of all studies that individuals were included regardless of disease severity or degree of chest wall rigidity, postural abnormalities or experiences of pain. There was no additional screening of individuals with COPD for underlying musculoskeletal dysfunction, postural evaluation or identification of musculoskeletal pain within the thoracic region in these studies. However, the intensive nature of the treatment suggests that it may be astute to explore the characteristics of those individuals who may achieve the most optimal outcomes. A range of tools to assess postural dysfunction and pain are available, have been validated in the COPD population [49,50], and could be applied to identify those who might benefit most from manual therapy treatments. 

In conclusion, musculoskeletal changes of altered chest wall mechanics, postural changes and pain are features of COPD, and it is important to identify treatment options that may address these abnormalities. Manual therapy techniques applied during a single treatment sessions have demonstrated some benefit in physiological parameters and symptoms. When applied in combination with exercise therapy or pulmonary rehabilitation, there has been mixed reports of improvements in selected measures of lung function and functional exercise capacity. However, the acceptance and role of manual therapy within clinical practice is limited by the labour and time intensity of individual treatment sessions, the lack of clarity of the optimal approach to treatment technique selection, the small participant numbers and the absence of long term follow up of clinical effects. Further examination to identify the characteristics of those with COPD who will most benefit from manual therapy, the optimal types of treatment approaches and their cost effectiveness, the ideal prescription and the longevity of effects is required. 

## Figures and Tables

**Table 1:** Techniques and prescription of manual therapy treatments in COPD.

Study Authors/Year	Participant Numbers	Description of Technique	Duration of Treatment	Outcome Measures	Findings
Engel et al. 2013 [34]	*N* = 15	**Soft tissue (ST)**: gentle massage, effleurage, friction and cross friction to intercostal, serratus anterior and posterior, rhomboid, trapezius, latissimus dorsi, erector spinae, quadratus lumborum and levator scapulae muscles. **Spinal Manipulation (SM)**: HVLA joint manipulation of thoracic inter-vertebral, costo-vertebral and costo-transverse joints. **Exercise (Ex)**: continuous walking on level surface for 6 min. **Group 1**: ST alone **Group 2**: ST + SM **Group 3**: ST + SM + Ex	**ST + SM** = 15–20 min **ST + SM + Ex** = 30 min 4 weeks of treatment, twice weekly	CRQ, pulmonary function, 6MWT	Increase in FVC and distance walked for ST + SM + Ex compared to ST only and ST + SM (*p* < 0.001). Improvement in dyspnoea with ST + SM and ST + SM + Ex compared to ST alone (*p* < 0.001).
Engel et al. 2016 [35]	***N* = 33**	**ST**: gentle effleurage, friction and cross-fibre friction to posterior chest wall, including intercostal, serratus anterior and posterior, rhomboid, trapezius, latissimus dorsi, erector spinae, quadratus lumborum and levator scapulae muscles. **SM**: graded delivery of HVLA joint manipulation of thoracic inter-vertebral, costo-vertebral and costo-transverse joints. **Pulmonary rehabilitation (PR)**: 24-week program, split into Intervention and Non-Intervention Phases. **Intervention**: 8 weeks Introduction—patients assessed for capacity to exercise, introduction to exercise and health education, then 8 weeks maintenance—gradual increase in exercise intensity to a level suitable for patient. **Non-intervention**: 8 weeks of no active intervention, patients instructed to exercise at their own discretion. **Group 1**: PR only **Group 2**: ST + PR **Group 3**: ST + SM + PR	**ST + SM** = 20 min, delivered twice a week from week 4 to week 12 of pulmonary rehabilitation.	Pulmonary function, SGRQ, HADS, 6MWT	Increase in FVC at 24 weeks with ST + SM + PR compared to PR only (*p* = 0.03). No significant difference in 6MWT, SGRQ or HADS between groups.
Noll et al. 2008 [40]		**Osteopathic Manipulative Treatment (OMT)**: treatment of specific somatic dysfunction, identified during examination, followed by seven standardised techniques: 1. ST massage of paraspinal muscles 2. Rib raising through traction applied to the rib angle, repeated until improved rib function was obtained 3. Diaphragm Release: ‘redoming’ the abdominal diaphragm—indirect myofascial release through contact of the diaphragmatic attachment near the thoracolumbar junction, and the abdominal epigastric area. The therapist rotated their hands in opposite directions until a release of tension or restriction was palpated. 4. Suboccipital decompression through outward and cephalad traction to the occipital condyles. 5. Thoracic inlet myofascial release by holding the tissue around the thoracic inlet until a relaxation or release of the tissue was palpated. 6. Pectoral traction by cephalad traction through the inferior border of the pectoral muscles, until release of upper respiratory muscle tension was palpated. 7. Thoracic lymphatic pump with activation by rhythmic pumping action, created by alternating pressure on the chest wall during inhalation and maintenance of some pressure during inspiration. On the fourth or fifth inhalation, therapist’s hands were removed during the first third of the inspiratory phase to create negative intrathoracic pressure. This cycle was repeated 3 times. **Sham Treatment**: Supine lying with light touch in same anatomical regions as OMT treatment.	**OMT** = Approximately 20 min. Single treatment session only.	Pulmonary function, Airway resistance and conductance, subjective report of effect on breathing.	Reduction in FEF_50%_ L/sec and FEF_25–75%_ L/sec with OMT compared to Sham (all *p* < 0.05). Increase in IC, RV, TLC and RV/TLC ratio with OMT vs. Sham (all *p* < 0.05). No other differences between groups.
Yelvar et al. 2016 [41]		**Technique**: Suboccipital decompression, cervical vertebral articulation gliding in the anterior/posterior plane, myofascial release of sternocleidomastoid, trapezius, intercostal and paravertebral muscles, gliding of sternoclavicular joint and thoracic vertebral articulations in the anterior/posterior direction, diaphragmatic release, rib raising, mobilization of scapulo-thoracic joint.	**MT** = myofascial release techniques applied each for 3–5 min, gliding techniques performed for 30 s and 5 times at each joint.	Pulmonary function, maximal inspiratory and expiratory pressures, HR, RR, SpO_2_, fatigue and dyspnoea perception according to Modified Borg rating of perceived exertion, subjective perception of ease of breathing.	Improvement in FEV_1_, FVC, VC following treatment (all *p* < 0.05). Increase in MIP (*p* = 0.03) and MEPS (*p* = 0.01) following treatment. Reduced HR and RR (*p* < 0.05), increased SpO_2_ (*p* = 0.01), reduced dyspnoea and fatigue (*p* = 0.01) and greater ease of breathing (*p* < 0.001).
Zanotti et al. 2012 [42]		**OMT**: Therapeutic application of manually guided forces to improve physiologic function and/or support homeostasis as identified through an examination of the thoracic outlet, spine, rib cage, thoracic and pelvic diaphragm, tentori-cerebellum and cranio-sacral evaluation. **PR**: 4-week exercise training, educational support, psychological counselling and nutritional intervention. Exercise training consisted of one session on the cycle ergometer and on cyclette, 5 days a week, for a total of 40 sessions. **Group 1**: OMT and PR **Group 2**: PR alone	**OMT** = 45 min, once a week for 4 weeks. Single treatment session.	6MWT, pulmonary function	Greater improvement in 6MWD with OMT + PR added to PR compared to PR alone (49 m (95% CI 17 to 81)). Reduction in RV with OMT + PR (−0.44L (95% CI 0.26 to 0.62)) compared to PR alone.
Rocha et al. 2015 [43]		**Manual Diaphragm Release Technique (MDRT)**: Hypothenar region and last three fingers of therapist’s hands were in contact with the underside of the costal cartilages of ribs 7 to 10. During the inspiratory phase, a gentle cepahald pull was applied, accompanying the physiological elevation of the ribs. In the expiratory phase, therapist contact was deepened towards the inner costal margin. On subsequent breaths, the therapist aimed to smoothly deepen contact and gain traction. **Sham treatment**: Manual contacts, duration, patient and therapist positioning were same as MDRT, but light touch only was applied to same anatomical landmarks, with no pressure or traction.	**MDRT** = 2 sets of 10 breaths, with a 1 min interval between sets. Six treatment sessions, 1 to 2 days apart during a 2 week period.	6MWT, diaphragmatic mobility, maximal inspiratory and expiratory pressures, chest wall and abdominal volumes using optoelectronic plethysmography	Cumulative increase in diaphragmatic mobility with MDRT: 18 mm (95% CI 8–28) compared to Sham. Increased in 6MWD by 22 m (95% CI 11 to 32) with MDRT. No difference in maximal respiratory pressures between treatments. Cumulative improvement in VC of 330 mL (95% CI 100 to 560) with MDRT compared to Sham.

Keys: ST, soft tissue; SM, spinal manipulation; HVLA, high volume low amplitude; Ex, exercise; CRQ, chronic respiratory questionnaire; FVC, forced vital capacity; FEV_1_, forced expiratory volume in one second; PR, pulmonary rehabilitation; SGRQ, St George’s Respiratory questionnaire; HADS, hospital anxiety and depression scale; 6MWT, 6 min walk test; OMT, osteopathic manipulative therapy; FEF_50%_, forced expiratory ratio at 50%; L/sec, litres per second; FEF_25–75%,_ forced expiratory ratio at 25–75%; IC, inspiratory capacity; RV, residual volume; TLC, total lung capacity; RV/TLC, residual volume to total lung capacity ratio; MDRT, manual diaphragm release technique; 6MWD, 6 min walk distance; CI, confidence interval; VC, vital capacity; MIP, maximal inspiratory pressure; MEP, maximal expiratory pressure; HR, heart rate; RR, respiratory rate; SpO_2_, percutaneous oxygen saturation.
